# Inter the future: a key opportunity for podiatry through inter-professional education

**DOI:** 10.1186/1757-1146-3-S1-O8

**Published:** 2010-12-20

**Authors:** Deborah Craddock

**Affiliations:** 1 University of Southampton, Southampton, Hampshire, UK

## Education conference theme

To show the vital way in which interprofessional education informs other health and social care professions of podiatrists’ roles and extending scope of practice. The importance of this input to the pre-qualifying curriculum cannot be underestimated to facilitate the integration of the podiatrist within the healthcare team.

## Introduction

Interprofessional education (IPE) and collaborative practice can play a significant role in mitigating challenges faced by global health care systems. To provide holistic care, health care professionals must be cognisant of podiatrists’ extending scope of practice. This national, evaluative study explored the influence of IPE at enhancing health and social work students’ awareness of podiatrists’ roles.

## Methods

Two prospective cross-sectional surveys were carried out in which a standardised questionnaire pack was administered to an independent volunteer sample of pre-registration health and social care students before an IPE initiative in 6 higher education institutions (HEIs) (Sample 1: n=1151), and on completion of an IPE initiative in 5 HEIs (Sample 2: n=1060). Steps were taken to promote the validity and reliability of the questionnaire pack and results were analysed statistically.

## Results

Findings revealed students’ knowledge of podiatrists’ roles was significantly better if they had participated in an IPE group with podiatry representation. This level of knowledge was influenced by institutional models of IPE across participating HEIs.

## Discussion

This study illuminates the key role of IPE and podiatry students to enhance awareness of podiatrists’ roles and extending scope of practice. The implications of these findings are explored and key recommendations are provided to facilitate the development of a collaborative practice-ready workforce.

This topic is highly relevant as the role of IPE and collaborative practice has been made explicit in the World Health Organisation’s (2010) Framework for Action to strengthen health systems.

This presentation will enable (i) participants to be aware of the important role IPE and podiatry students play in enabling future health and social care professionals to be aware of the profession’s scope of practice; & (ii) curriculum developers to be aware of factors to consider when developing IPE curricula (pre- or post qualifying).

